# Atorvastatin and almonertinib-induced myopathy in a polypharmacy context: a case report

**DOI:** 10.3389/fcvm.2026.1805200

**Published:** 2026-05-14

**Authors:** Jifang Zhou, Lixia Yu, Huimin Xu

**Affiliations:** 1 Department of Pharmacy, Linping Campus, The Second Affiliated Hospital of Zhejiang University School of Medicine, Hangzhou, Zhejiang, China; 2 Department of Pharmacy, Yuecheng District People’s Hospital of Shaoxing, Shaoxing, Zhejiang, China; 3 The Second Affiliated Hospital of Zhejiang University School of Medicine, Hangzhou, Zhejiang, China

**Keywords:** adverse drug reactions (ADR), creatine kinase, epidermal growth factor receptor tyrosine kinase inhibitor (EGFR-TKI), myopathy, statin

## Abstract

**Background:**

Statin-induced myopathy is a common adverse effect and the risk of myopathy is increased by interacting drugs, usually inhibitor of CYP3A4. Almonertinib is a novel third-generation epidermal growth factor receptor tyrosine kinase inhibitor (EGFR-TKI) and is a moderately sensitive substrate of CYP3A4. Here we report a case of myopathy probably caused by concomitant use of atorvastatin and almonertinib.

**Case summary:**

An 89-year-old male patient with coronary heart disease and lung cancer was admitted to the hospital due to worsened chest tightness and shortness of breath for one week. He had been taking almonertinib for 26 months, hybutimibe for 3 months and alirocumab for 1 month prior to admission. Atorvastatin was added because the patient failed to reach the target cholesterol concentration. He presented with myasthenia and markedly elevated his serum creatine kinase (CK) (2,103 U/L; normal range <164 U/L) 1 day later. Drug-induced myopathy was diagnosed with a probable drug-drug interaction between atorvastatin and almonertinib. After discontinuation of the two drugs, the patient's CK returned to normal.

**Conclusion:**

Almonertinib is a moderately sensitive substrate of CYP3A4, thereby may elevate statin concentration and increase the likelihood of developing myopathy. For patients who need to take almonertinib and cholesterol-lowering drugs simultaneously, proprotein convertase subtilisin/kexin type 9 (PCSK9) inhibitors and cholesterol absorption inhibitors (such as ezetimibe and hybutimibe) can be considered instead of atorvastatin for the reduction of cardiovascular events.

## 1 Introduction

Cardiovascular disease (CVD) and cancer are the two major causes of death worldwide (1). According to the latest global health estimates, non-communicable diseases (NCDs) are responsible for approximately 74% of all deaths worldwide. Of the estimated 17 million premature deaths occurring before the age of 70, NCDs account for more than 80%, with cardiovascular diseases, cancers, chronic respiratory diseases, and diabetes remains the leading causes of mortality among those aged 30–70 (2). Of every 10 premature deaths from non-communicable diseases, 4 are due to CVD and 3 to cancer. 3-hydroxy-3-methylglutaryl-CoA (HMG-CoA) reductase inhibitors (statins) are currently recommended for secondary prevention of CVD (3). The most common adverse drug reactions (ADR) to statins include myopathy and elevated serum aminotransferase levels, which occur in about 5% and 0.5%–2.0% of users, respectively (4).

Almonertinib is a novel third-generation epidermal growth factor receptor tyrosine kinase inhibitor (EGFR-TKI) targeting EGFR-TKI-sensitive gene mutations and T790M resistance mutations (5). Almonertinib was approved in 2020 for the treatment of patients with EGFR T790M mutation-positive non-small cell lung cancer (NSCLC) who had progressed or advanced after other EGFR-TKI treatment, and was subsequently approved in 2021 for the first-line treatment of patients with EGFR T790M mutation-positive advanced NSCLC (6). The most common adverse reactions to almonertinib include elevated CK, mild rash, and diarrhea (7). In clinical studies, elevated CK occurred in 7.0% of patients at a dose level of 110 mg (8).

CVD and cancer are common and serious diseases. As cancer treatment and survival rates improve, CVD is increasingly emerging as a subsequent clinical condition in this patient population (9). The risk of statin-induced myopathy usually increases with the number of interacting drugs, usually CYP3A4 inhibitors (10). Among the drugs, almonertinib is a moderately sensitive substrate of CYP3A4 (11), which theoretically increases the risk of myopathy when combined with statins. While the drug label for almonertinib warns of potential risks when combined with statins, detailed clinical documentation of this specific interaction—particularly its onset characteristics and management in ultra-elderly patients—remains scarce in the literature. Here, we report a case of myopathy potentially induced by the concurrent use of almonertinib and atorvastatin.

## 2 Case presentation

### 2.1 Chief complaints

An 89-year-old Asian man presented with a 3-month history of chest pain and a 1-week history of chest tightness and shortness of breath.

### 2.2 History of present illness

Three months ago, the patient was hospitalized in another hospital for a follow-up examination of lung cancer and suddenly experienced chest pain. At that time, “acute myocardial infarction” was considered, and coronary angiography was performed, which indicated occlusion of the anterior descending branch, occlusion of the right coronary sub-branch, and 80%–90% stenosis of the circumambulation branch. A stent was implanted in the right coronary artery, and the patient's chest pain improved after the operation.

The patient reported a 1-week history of progressively worsening chest tightness and dyspnea without identifiable triggers. His symptoms, which initially were mild and overlooked, gradually intensified; however, he denied any syncope, cough, or hemoptysis. He was hospitalized and managed with conservative measures including diuretics, anti-infectives, and potassium supplementation, resulting in symptomatic alleviation. His mental status, diet, sleep, and bowel habits remained normal throughout the illness, with no significant weight change.

### 2.3 History of past illness

1. The patient had a history of schizophrenia for more than 40 years and was taking olanzapine 10 mg once daily and zopiclone 7.5 mg once daily for a long time.
2. The patient had a 4-year history of left upper lung adenocarcinoma with brain metastasis. Genetic testing of a lung biopsy revealed an EGFR Ex19 DEL mutation but was negative for ALK and ROS1 rearrangements. He was managed non-surgically with an initial regimen of icotinib (125 mg three times daily) and bevacizumab (450 mg IV every 3 weeks). Two years ago, follow-up imaging revealed disease progression, leading to a switch in therapy to almonertinib (110 mg once daily) while continuing bevacizumab.
3. Three months ago, during a routine lung cancer follow-up at another hospital, the patient was diagnosed with acute myocardial infarction. A coronary angiogram revealed occlusion of the anterior descending branch, secondary occlusion of the right coronary artery, and 80%–90% stenosis of the retrograde branch. A stent was implanted in the right coronary artery, which alleviated his chest pain. During this hospitalization, the patient was prescribed atorvastatin 20 mg nightly. Subsequently, he developed elevated creatine kinase (CK) and liver enzymes. Both atorvastatin and almonertinib were discontinued, and his CK and liver enzyme levels normalized 16 days later. Upon discharge, his medication regimen was established as follows: aspirin 100 mg once daily, ticagrelor 90 mg twice daily, alirocumab 75 mg subcutaneously every two weeks, pantoprazole 40 mg once daily, metoprolol 12.5 mg once daily, and sacubitril/valsartan 50 mg twice daily.
4. The patient has no history of drug allergy or adverse drug reactions.

### 2.4 Personal and family history

The patient reported no significant family history of hereditary, infectious, psychiatric, or neoplastic diseases, and denied any personal history of smoking, alcohol consumption, chronic medication use, or exposure to toxins.

### 2.5 Physical examination

On admission, vital signs were stable: Temperature was 36.7 °C, pulse 69–80 beats per minute, respiratory rate 18–20 breaths per minute, and blood pressure 109/63 mmHg. Physical exam revealed an alert and cooperative patient without scleral icterus. Cardiovascular exam showed a regular rhythm without murmurs. Auscultation of the lungs revealed coarse breath sounds with bilateral lower-lobe wet rales. The abdomen was soft and non-tender, with no hepatosplenomegaly; Murphy's sign and shifting dullness were negative. There was no peripheral edema, and pathological reflexes were absent. Allen's test was positive.

### 2.6 Laboratory examinations

The results of laboratory examinations revealed a mild increase in high-sensitivity cardiac troponin T (hs-cTnT) 0.075 ng/mL (normal range <0.014 ng/mL), and a significant increase in N-terminal pro-brain natriuretic peptide (NT-proBNP) 34,209 pg/mL (normal range <900 pg/mL), CK 495 U/L (normal range <164 U/L), with normal creatine kinase isoenzyme (CK-MB), alanine aminotransferase (ALT), and aspartate aminotransferase (AST). Therefore, he continued to maintain the original medication regimen after admission.

On Day 2 of admission, atorvastatin 20 mg once daily in the evening was added because the laboratory tests revealed his low-density-lipoprotein (LDL) cholesterol was 2.57 mmol/L.

**Figure 1:**
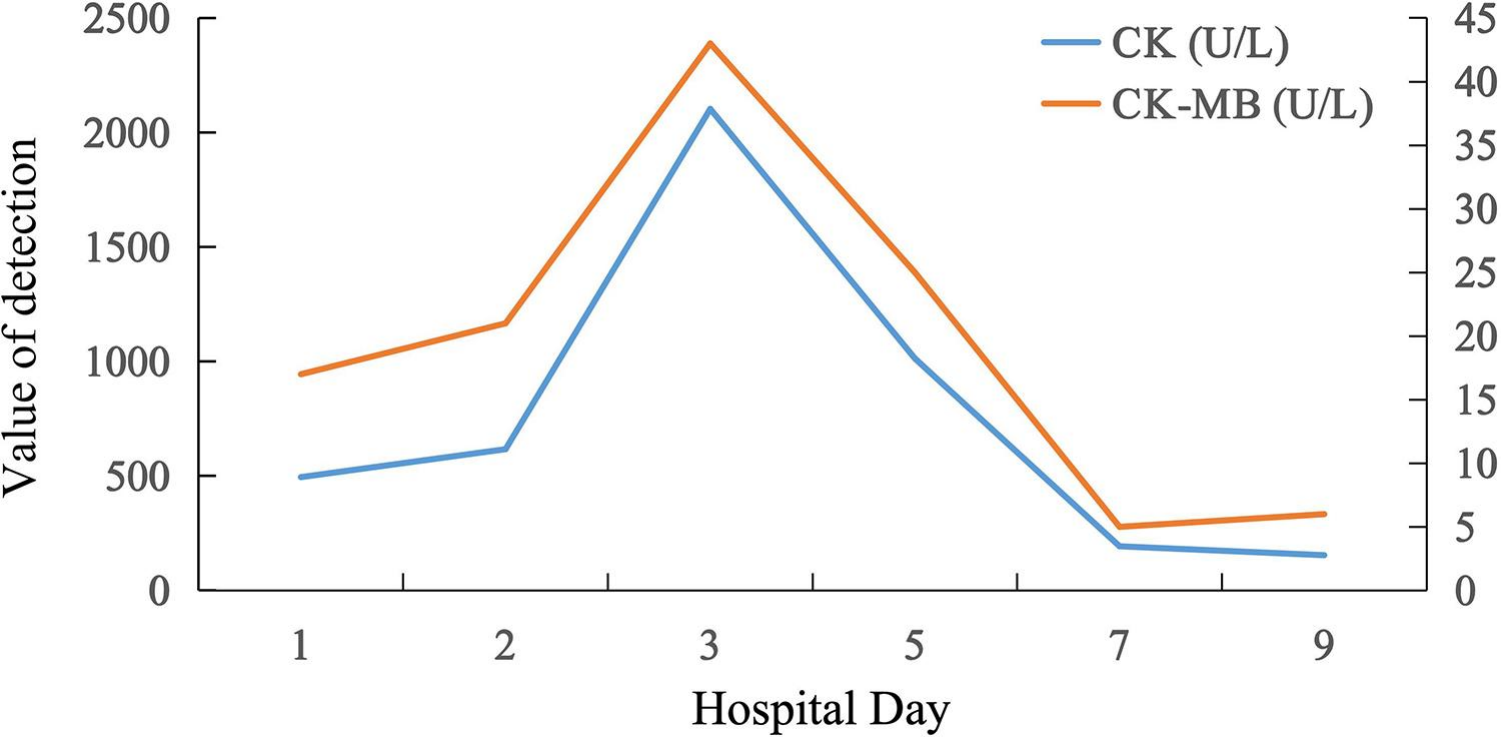
The trends of serum creatine kinase (CK), creatine kinase-MB (CK-MB) levels during hospitalization.

On day 3, laboratory tests revealed slightly elelvated hs-cTnT 0.124 ng/mL, markedly elevated CK 2103 U/L (Figure 1) and CK-MB 43 U/L(normal range <24 U/L). There were no symptoms of myalgia, but the patient felt muscle weakness. He had no excessive exercise or trauma.

On day 4, laboratory tests showed a concomitant rise in serum creatinine (Scr) from a baseline of 184 to 219.3 μmol/L, indicating a mild acute kidney injury (AKI) superimposed on chronic renal insufficiency. Following the cessation of the suspected medications, the Scr level gradually declined and stabilized at 180 μmol/L on Day 8.

### 2.7 Diagnostic examination

The patient was admitted to the hospital with an electrocardiogram showing sinus rhythm and first-degree atrioventricular block. The echocardiogram showed post-percutaneous coronary intervention (post-PCI), abnormal left ventricular myocardial segmental motion, left atrial enlargement, left heart insufficiency, and the LVEF was 30%–40%.

### 2.8 Imaging examinations

The patient was admitted to the hospital with a CT of the lungs showing a parafoveal lesion in the left interlobar fissure, and a CT plain scan of the abdomen did not show any obvious signs of urgency.

### 2.9 Final diagnosis

Heart failure, Coronary heart disease, Lung cancer, Schizophrenia, Myopathy.

### 2.10 Treatment

Almonertinib and atorvastatin were discontinued on the 4th day of admission, and ezetimibe 10 mg once daily and evolocumab injection 140 mg inject subcutaneously every 2 weeks were given to lower lipids. The patient's CK levels decreased after discontinuation and returned to normal on the 7th day of admission (Figure 1).

### 2.11 Outcome and follow-up

Due to the risk of recurrent CK elevation, statin therapy was not reinitiated. Furthermore, given the progression of his lung cancer, almonertinib was also withheld. The patient remained asymptomatic, reporting no chest tightness, pain, chills, or fever, and was subsequently discharged in this stable condition.

## 3 Discussion

**Figure 2:**
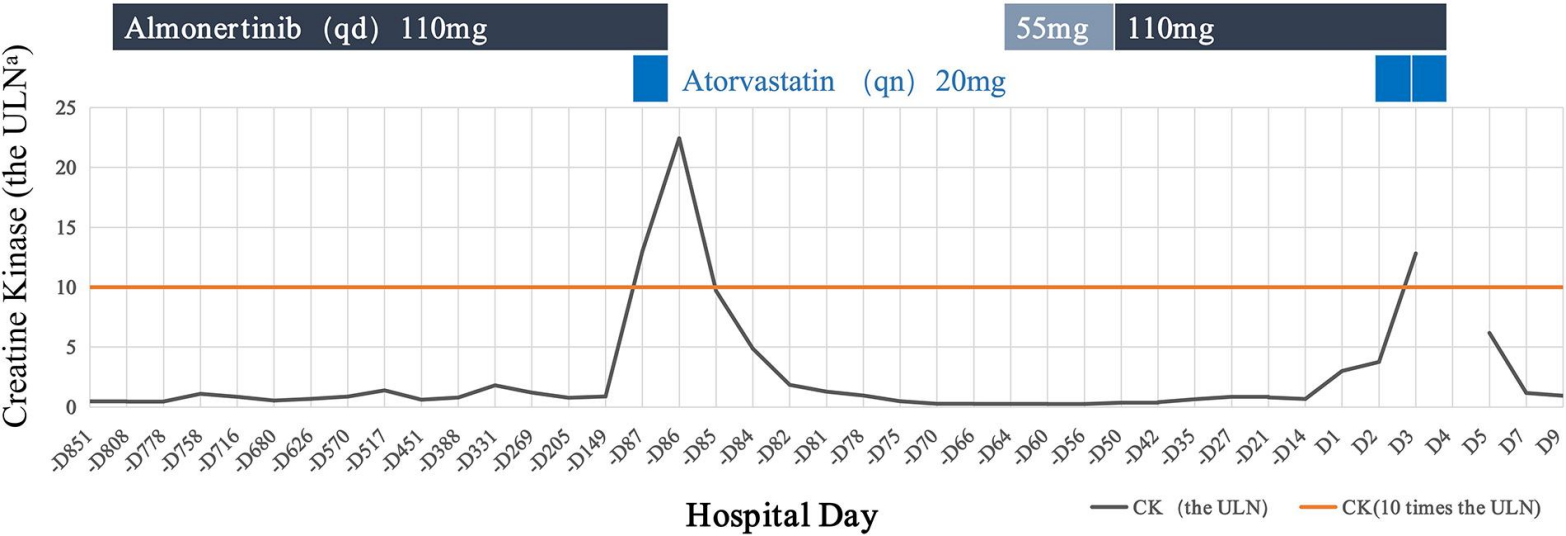
Trends in the patient's serum creatine kinase (CK) levels during treatment with almonertinib and atorvastatin.

Although the potential for CK elevation is noted in the almonertinib package insert, the clinical severity and the exceptionally rapid onset (within 24 h) observed in this 89-year-old patient underscore a much more potent synergistic effect than previously characterized. This case report describes a suspected drug-drug interaction between almonertinib and atorvastatin, leading to a significant elevation in creatine kinase (CK) and myopathy in an 89-year-old man. The patient recalled that he had suffered an acute myocardial infarction 3 months ago in another hospital, and had taken atorvastatin 20 mg once daily in the evening. His CK and liver enzymes were dramatically increased 1 day after the patient used the statin (Figure 2). The peak concentration of CK was 6953 U/L (normal range: 50–310 U/L), ALT was 1547 U/L (normal range: 9–50 U/L), and AST was 2041 U/L (normal range: 15–40 U/L). At that time, atorvastatin and almonertinib were discontinued, and hybutimibe (a novel selective cholesterol absorption inhibitor developed in China) 10 mg once daily and alirocumab injection 75 mg inject subcutaneously every 2 weeks were added. The patient's liver enzymes and CK returned to normal 16 days after discontinuation the two drugs. A small dose of almonertinib (55 mg once daily) was added on 22 days after discontinuation the two drugs and gradually increased to 110 mg once daily, during which the liver enzymes were not abnormal and the CK had a mild elevation with asymptomatic, but not more than 3 times the upper limit of normal (ULN). The Naranjo Adverse Drug Reaction (ADR) Probability Scale score of atorvastatin was 7 and the score of almonertinib was 3 (Table 1). The score of the SAMS-CI scale was 9 (Table 2). The above suggests that atorvastatin is more likely the cause of elevated CK levels in patients.

**Table 1:** Naranjo score results of patient-related adverse reactions caused by almonertinib and atorvastatin calcium tablets.

Criteria	Yes	No	Don't know	Score	
				Atorvastatin	Almonertinib
1. Are there previous conclusive reports on this reaction?	+1	0	0	1	1
2. Did the adverse reaction appear after the suspected drug was administered?	+2	−1	0	2	2
3. Did the adverse reaction improve when the drug was discontinued or a specific antagonist was administered?	+1	0	0	1	1
4. Did the adverse reaction reappear when the drug was readministered?	+2	−1	0	2	−1
5. Are there alternative causes that could on their own have caused the reaction?	−1	+2	0	−1	−1
6. Did the reaction reappear when a placebo was given?	−1	+1	0	0	0
7. Was the drug detected the blood (or other fluids) in concentrations known to be toxic?	+1	0	0	0	0
8. Was the reaction more severe when the dose was increased or less severe when the dose was decreased?	+1	0	0	0	0
9. Did the patient have a similar reaction to the same or similar drug in any previous exposure?	+1	0	0	1	0
10. Was the adverse event confirmed by any objective evidence?	+1	0	0	1	1
SCORING				7	3

Tips: ≥9 = definite; 5–8 = probable; 1–4 = possible; ≤0 = doubtful.

**Table 2:** Statin-associated muscle symptoms clinical index (SAMS-CI).

If one regimen of statin involved (questions regarding this regimen)			Enter score
A. Location and pattern of muscle symptoms (If more than one category applies, record the highest number.)	Symmetric, hip flexors or thighs	3	1
	Symmetric, calves	2	
	Symmetric, proximal upper extremity	2	
	Asymmetric, intermittent or not specific to an area	1	
B. Timing of muscle symptom onset in relation to starting statin regimen	<4 weeks	3	3
	4–12 weeks	2	
	>12 weeks	1	
C. Timing of muscle symptom improvement after withdrawal of statin (If patient is still taking statin, stop regimen and monitor symptoms.)	<2 weeks	2	2
	2–4 weeks	1	
	No improvements after 4 weeks	0	
Rechallenge the patient with a statin regimen, (even if same statin compound or regimen as above) then complete final question:			
D. Timing of recurrence of similar muscle symptoms in relation to starting second regimen	<4 weeks	3	3
	4–12 weeks	1	
	>12 weeks or symptoms did not reoccur	0	
Total: All four scores above must be entered before totalling			9

Tips: 2–6 = Unlikely; 7–8 = Possible; 9–11 = Probable.

Considering the patient's previous medication history, drug-induced myopathy probably caused by a drug-drug interaction between atorvastatin and almonertinib was diagnosed. Almonertinib and atorvastatin were discontinued on the 4th day of admission, and ezetimibe 10 mg once daily and evolocumab injection 140 mg inject subcutaneously every 2 weeks were given to lower lipids. The patient's CK levels decreased after discontinuation and returned to normal on the 7th day of admission (Figure 1). Considering the risk of recurrent CK elevation, statins were not prescribed to the patient again. The patient was not given almonertinib back because his lung cancer progressed.

Myopathy is characterized by unexplained muscle pain or weakness with CK concentrations exceeding 10 times the ULN, and occurs in 1 patient per 10,000 patients annually (12). Through detailed medical history collection and auxiliary examinations, common non-drug-induced CK elevation factors such as overexertion, trauma, viral infection, and metabolic abnormalities were ruled out, thus focusing the investigation on drug interference (13). Notably, while olanzapine has been reported to potentially influence CK levels, it was excluded as a primary causative factor in this case. The patient had maintained a stable dose of olanzapine for over 5 years with consistently normal baseline CK values. The acute surge and subsequent resolution of CK synchronized precisely with the introduction and withdrawal of the atorvastatin-almonertinib combination, while olanzapine therapy remained unchanged throughout.

Regarding the other medications in the patient's regimen, the impact of ticagrelor and pantoprazole on the CYP3A4 enzyme must be carefully evaluated. Both ticagrelor and atorvastatin are substrates of CYP3A4, and ticagrelor is also a weak inhibitor of this isoenzyme and a known P-glycoprotein (P-gp) inhibitor. Theoretically, this combination could increase statin exposure. However, in this case, the patient had tolerated a stable dose of ticagrelor and pantoprazole for over 3 months post-infarction with consistently normal CK levels. This suggests that while these agents may have occupied a portion of the metabolic “reserve” of the CYP3A4 pathway, they were not the direct triggers of acute muscle injury. The acute reduction in muscle cell stability was likely the result of a “multi-hit” pharmacological event. The chronic presence of ticagrelor and almonertinib essentially narrowed the metabolic window, creating a state of “metabolic crowding.” When atorvastatin was re-introduced, this competition led to a rapid, disproportionate spike in its plasma concentration. Synergistically, the biochemical destabilization of the sarcolemma by high-dose statin exposure combined with almonertinib's inhibition of EGFR-mediated muscle repair, led to the precipitous skeletal muscle toxicity observed. The precise temporal correlation—where CK levels spiked only upon atorvastatin introduction and resolved upon its withdrawal—identifies the atorvastatin-almonertinib-ticagrelor interplay as the key driver of this adverse event, rather than isolated toxicity from long-term medications.

**Figure 3:**
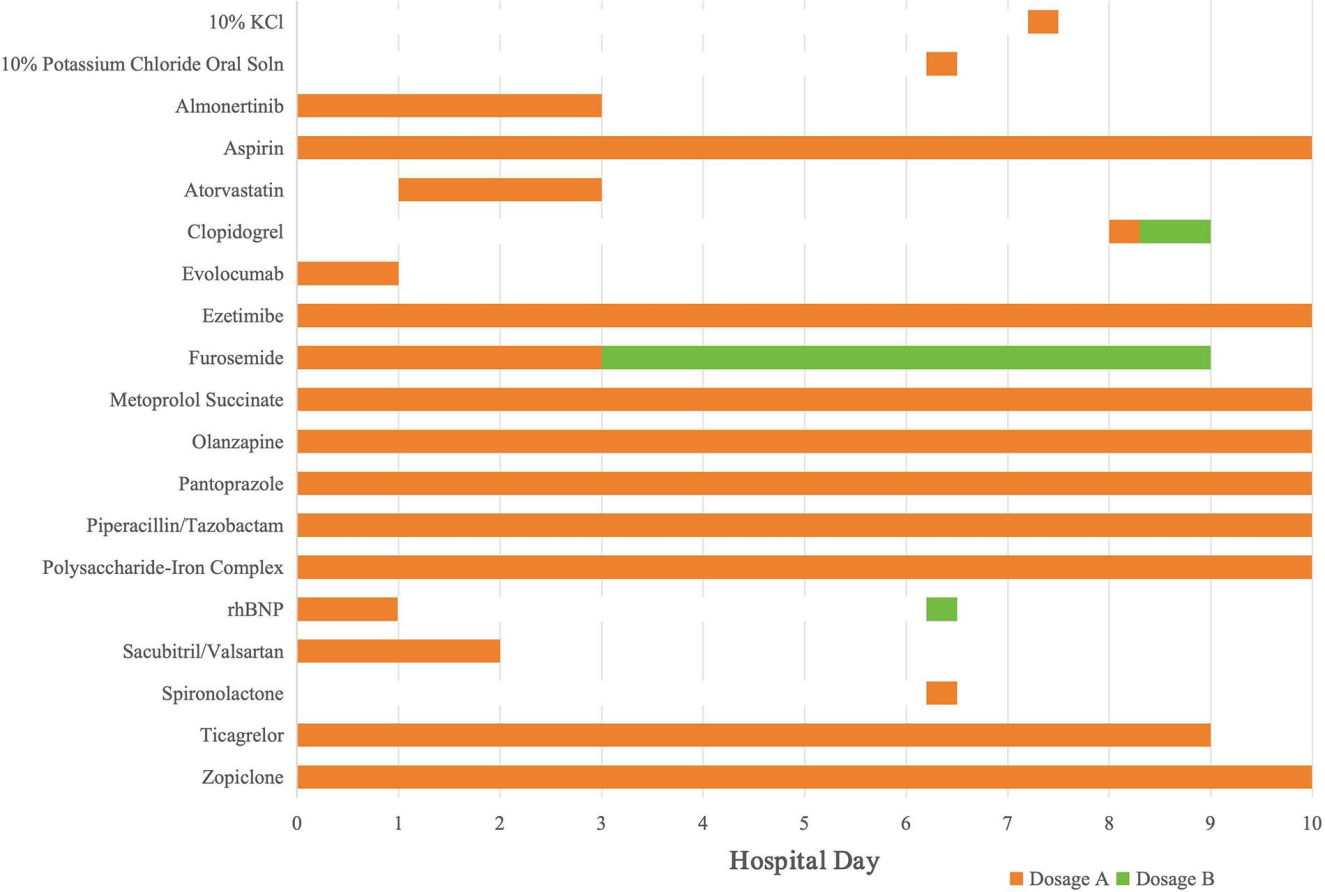
Timeline of pharmacological interventions during hospitalization.

The elevation of biomarkers in this case should be interpreted as drug-induced skeletal muscle toxicity rather than myocardial injury associated with chronic coronary syndrome (CCS). Although both CK and CK-MB peaked on Day 3 (2,103 U/L and 43 U/L, respectively), the CK-MB/CK ratio was approximately 2.04%. In clinical practice, a ratio below 5% is a hallmark of skeletal muscle origin, as myocardial infarction typically presents with a ratio exceeding 5%–10%. Furthermore, the acute “spike-and-fall” trajectory of these enzymes showed a precise temporal correlation with the re-introduction (Day 2) and subsequent discontinuation (Day 4) of atorvastatin, as visualized in the medication timeline (Figure 3). Before the onset of acute myocardial infarction, the patient had been taking almonertinib for 26 months, with assessments every 2 months, during which time CK levels were slightly elevated, asymptomatic, but not more than 3 times the ULN. After taking atorvastatin 20 mg once, CK levels increased to more than 20 times the ULN. After withdrawal of almonertinib and atorvastatin, CK levels returned to normal. After re-addition of almonertinib, there was no significant increase in CK. However, during the current admission, atorvastatin was reintroduced on Day 2, which immediately led to a second spike in CK levels exceeding 10 times the ULN by Day 3. The patient did not have symptoms of myalgia, but felt weak. On the sixth day after discontinuing both medications, his creatine phosphokinase returned to normal. Based on his past medication history, we hypothesized that the patient's myopathy was caused by the combination of atorvastatin and almonertinib.

Although the mechanism by which almonertinib induces myopathy is not yet clear, an *in vitro* study reported that EGFR plays a critical role in the differentiation of myoblasts. Therefore, almonertinib may affect myoblasts by inhibiting T differentiation and blocking the EGFR pathway, leading to skeletal muscle injury (14).

The mechanism of myopathy caused by statins is not fully understood. While the pathophysiology of SAMS involves multiple factors—including impaired mitochondrial function, depleted coenzyme Q10, and destabilized calcium homeostasis—advanced age remains a critical risk factor (15–17). In this case, the onset of severe myopathy was unusually rapid, occurring within 24 h of atorvastatin re-introduction. This clinical trajectory strongly suggests a potent pharmacokinetic interaction rather than isolated statin intolerance. Almonertinib and atorvastatin are primarily metabolized by CYP3A4, and there may be metabolic drug interactions between the two, causing an increase in atorvastatin blood drug concentrations (18, 19). Luo et al. (20) found that on the one hand, EGFR-TKIs can reduce the expression of low-density lipoprotein receptor (LDLR) by blocking SREBP-1-dependent EGFR signaling pathway, which may reduce the utilization of cholesterol; on the other hand, the application of atorvastatin can inhibit cholesterol synthesis and limit tumor growth by inhibiting the synthesis of cholesterol to limit tumor growth, which means that EGFR-TKI and statin can act together on LDLR to influence each other's efficacy.

The temporal relationship in this case provides strong evidence for a drug interaction rather than an isolated toxicity. The patient had tolerated almonertinib monotherapy for over 2 years without adverse effects; however, within 24 h of restarting atorvastatin, the patient developed acute severe myopathy, characterized by CK levels exceeding 10 times ULN. While both drugs are independently associated with muscle toxicity, the rapid deterioration of clinical symptoms observed here suggests a synergistic effect. Since both almonertinib and atorvastatin are major substrates of the CYP3A4 enzyme, competitive inhibition could lead to an acute increase in plasma statin concentrations. Simple use of almonertinib or atorvastatin cannot explain the current serious adverse reactions. Therefore, we believe that atorvastatin is the main cause of myopathy in the patient, and almonertinib is the secondary cause.

Given the patient's advanced age and pre-existing renal insufficiency, he was highly susceptible to pigment-induced nephropathy—a life-threatening complication of rhabdomyolysis. Although a transient Scr elevation occurred (184–219.3 μmol/L), prompt drug withdrawal prevented progression from Stage 1 AKI to overt renal failure or the need for dialysis. This highlights the necessity of vigilant renal monitoring in ultra-elderly patients experiencing drug-induced myotoxicity.

The transition from the pre-admission regimen (alirocumab/hybutimibe) to evolocumab and ezetimibe was a therapeutic substitution based on the hospital's drug formulary. As these agents share the same pharmacological classes and mechanisms of action with equivalent efficacy, the switch ensured treatment continuity. Detailed pharmacological specifications, including dosages and durations for all treatments, are provided in the medication list (Table 3).

**Table 3:** List of prescribed medications and medication administration timeline during hospitalization.

Drug name	Dosage & route	Timing	Drug name	Dosage & route	Timing
Aspirin	100 mg PO QD	D1–D10	Almonertinib	110 mg PO QD	D1–D3
Ticagrelor	90 mg PO BID	D1–D9	Evolocumab	140 mg SC Q2W	D1
Atorvastatin	20 mg PO QN	D2–D3	Ezetimibe	10 mg PO QN	D1–D10
Pantoprazole	40 mg PO QD	D1–D10	Furosemide	20 mg IV bolus QD	D4–D10
Furosemide	20 mg IV bolus BID	D1–D4	Polysaccharide-iron complex	0.15 g PO QD	D1–D10
Metoprolol succinate	12.5 mg PO QD	D1–D10	10% Potassium chloride oral soln	20 mL PO Once	D7
Sacubitril/Valsartan	50 mg PO BID	D1–D2	Spironolactone	20 mg PO Once	D7
0.9% NS	100 mL IV drip Q12H	D1–D10	0.9% NS	50 mL via syringe pump once	D7
Piperacillin/Tazobactam	4.5 g IV drip Q12H		rhBNP	0.5 mg via syringe pump once	
0.9% NS	50 mL IV Pump QD	D1–D2	0.9% NaCl	250 mL IV drip once	D8
rhBNP	0.5 mg IV Pump QD		10% KCl	5 mL IV drip once	
Olanzapine	10 mg PO QN	D1–D10	Clopidogrel	300 mg PO once	D9
Zopiclone	7.5 mg PO QN	D1–D10	Clopidogrel	75 mg PO QD	D9–D10

In this case, the inadvertent re-exposure resulted from fragmented medical records across different institutions and an incomplete medication history provided by the patient. This underscores the systemic challenges of ADR monitoring in real-world practice and highlights the critical necessity for meticulous medication reconciliation, particularly in elderly patients with complex polypharmacy. The management of SAMS requires a stratified strategy, from dose adjustment to a complete transition to non-statin therapy (21). In this case, the risk of progression to rhabdomyolysis or pigment-induced acute kidney injury was reduced by immediately identifying hypercreatine kinaseemia and subsequently discontinuing almonertinib and atorvastatin. This proactive medication adjustment underscores the importance of rigorous biochemical monitoring in elderly patients receiving complex multidrug regimens, ultimately ensuring a good clinical outcome.

## 4 Conclusions

In conclusion, the time relationship observed in this case suggests that almonertinib may enhance statin-induced muscle toxicity through pharmacokinetic interactions. To reduce the risk of serious adverse events, we recommend that patients taking almonertinib, especially elderly patients with multiple complex diseases, have their lipid-lowering regimen adjusted and switched to non-statin drugs, such as cholesterol absorption inhibitors or PCSK9 inhibitors. Further research is necessary to elucidate the specific mechanisms of this interaction.

## Data Availability

The original contributions presented in the study are included in the article/Supplementary Material, further inquiries can be directed to the corresponding author.
