# Supplementary material for: Atorvastatin and almonertinib-induced myopathy in a polypharmacy context: a case report
Source: Front Cardiovasc Med. 2026 May 14;13:1805200. doi: 10.3389/fcvm.2026.1805200 (PMC13215797; doi:10.3389/fcvm.2026.1805200)
Supplement: Supplementary file 1 [file Table1.pdf]

Supplementary Table S1: List of prescribed medications and medication administration timeline during hospitalization.

**Inpatient Medication List**		
**Drug Name**	**Dosage & Route**	**Timing**
Aspirin	100mg PO QD	D1-D10
Ticagrelor	90mg PO BID	D1-D9
Atorvastatin	20mg PO QN	D2-D3
Pantoprazole	40mg PO QD	D1-D10
Furosemide	20mg IV bolus BID	D1-D4
Metoprolol Succinate	12.5mg PO QD	D1-D10
Sacubitril/Valsartan	50mg PO BID	D1-D2
Polysaccharide-Iron Complex	0.15g PO QD	D1-D10
0.9% NS<br>rhBNP	50mL IV Pump QD<br>0.5mg IV Pump QD	D1-D2
Olanzapine	10mg PO QN	D1-D10
Zopiclone	7.5mg PO QN	D1-D10
Almonertinib	110mg PO QD	D1-D3
Evolocumab	140mg SC Q2W	D1
Ezetimibe	10mg PO QN	D1-D10
Furosemide	20mg IV bolus QD	D4-D10
0.9% NS	100mL IV drip Q12H	D1-D10

Piperacillin/Tazobactam	4.5g IV drip Q12H	
10% Potassium Chloride Oral Soln	20mL PO Once	D7
Spironolactone	20mg PO Once	D7
0.9% NS<br>rhBNP	50mL via Syringe Pump Once<br>0.5mg via Syringe Pump Once	D7
0.9% NaCl<br>10% KCl	250mL IV drip Once<br>5mL IV drip Once	D8
Clopidogrel	300mg PO Once	D9
Clopidogrel	75mg PO QD	D9-D10

Discharge Medication List

**Drug Name**	**Dosage & Route**
Aspirin	100mg PO QD
Clopidogrel	75mg PO QD
Pantoprazole	40mg PO QD
Ezetimibe	10mg PO QD
Evolocumab	140mg SC Q14D
Metoprolol Succinate	12.5mg PO QD
Olanzapine	10mg PO QN
Zopiclone	7.5mg PO QN
Cefdinir	100mg PO BID
